# The prognostic importance of the number of metastases in pulmonary metastasectomy of colorectal cancer

**DOI:** 10.1186/s12957-015-0621-7

**Published:** 2015-07-25

**Authors:** Jong Ho Cho, Seok Kim, Mi Namgung, Yong Soo Choi, Hong Kwan Kim, Jae Ill Zo, Young Mog Shim, Jhingook Kim

**Affiliations:** Department of Thoracic and Cardiovascular Surgery, Samsung Medical Center, Sungkyunkwan University School of Medicine, 50, Ilwon-dong, Gangnam-gu, Seoul 135-710 South Korea

**Keywords:** Multiple pulmonary nodules, Metastasectomy, Colorectal cancer

## Abstract

**Background:**

The presence of multiple metastatic pulmonary nodules is a predictor of poor survival after pulmonary metastasectomy. However, there is a paucity of data addressing the exact number of pulmonary metastases over which prognosis becomes grave. The aim of our study is to investigate the prognosis of pulmonary metastasectomy from colorectal cancer (CRC) depending on the number of pulmonary metastases.

**Methods:**

Patients who had undergone pulmonary metastasectomy for CRC between November 1994 and December 2013 were included. Survival and recurrence patterns were analyzed with regard to the number of pulmonary metastases. Patients were divided into three groups depending on the number of pulmonary metastases that were detected by the final pathologic report: group I—single metastasis; group II—2–3 metastases; and group III—4+ metastases.

**Results:**

A total of 615 patients who had undergone pulmonary metastasectomy from colorectal cancer were included. The median follow-up period was 31 months (range 2–211 months). The median disease-free interval (DFI) from the time of the primary operation for colorectal cancer was 20 months (range 0–209 months). There were 414 patients in group I (single metastasis), 159 in group II (2–3 metastases), and 42 in group III (4+ metastases). The overall 5-year survival rate was 64.2 %. The 5-year survival rates in groups I, II, and III were 70.0, 56.2, and 33.7 %, respectively (group I vs. II, *p* < 0.001; group II vs. III, *p* = 0.012). The 5-year recurrence-free rates were 39.5, 30.6, and 8.5 % in groups I, II, and III, respectively (group I vs. II, *p* < 0.001; group II vs. III, *p* = 0.056). Multivariable analysis revealed that age, multiple pulmonary nodules, thoracic lymph node metastasis, and adjuvant chemotherapy are independent predictors of survival.

**Conclusions:**

The overall survival and recurrence after pulmonary metastasectomy for CRC is dependent on the number of metastases. Surgical treatment can be offered to patients with three or fewer pulmonary metastases. However, more meticulous patient selection is required to decide whether a surgical approach is feasible in patients with four or more pulmonary metastases.

## Background

Approximately half of the patients who undergo resection of colorectal cancer (CRC) will develop metastatic disease and 5–15 % of those eventually develop lung metastases [1, 2]. Pulmonary metastases from CRC result from systemic tumor spread. Fortunately, the surgical resection of lung metastases can be curative in select patients. In the presence of pulmonary metastases, the treatment of choice depends on an assessment of resectability. There are several proposed prognostic factors that are related to survival and tumor recurrence after CRC pulmonary metastasectomy. The number of pulmonary metastases is negatively correlated to survival after pulmonary metastasectomy. Several studies have reported that patients with solitary lesions consistently have higher survival rates than do those with multiple lesions [3–5]. However, there is no known cutoff number of pulmonary metastases that influences metastasectomy success.

Our strategy in metastatic CRC has been to offer surgery as long as resectability and operability can be guaranteed, regardless of the number of pulmonary metastases. We reviewed prior cases to investigate the prognosis of pulmonary metastasectomy depending on the number of pulmonary metastases.

## Methods

### Patients and data collection

Data were collected on patients who underwent lung metastasectomy (from CRC) between November 1994 and December 2013. The patients’ baseline characteristics and treatment modalities were obtained from the medical records. Patients were included if they had the following: (1) complete resection for cure or control of primary CRC, (2) no extrapulmonary metastasis that precluded resection, and (3) a follow-up chest CT or PET-CT scan at our institution at least twice after surgery. Patients were excluded if there was no available radiologic information for surveillance after pulmonary metastasectomy.

Based on the analyses of pulmonary metastasectomy by the International Registry of Lung Metastases, we divided patients into three groups according to the number of pulmonary metastases: group I—single metastasis; group II—2–3 metastases; and group III—4+ metastases [6]. The “number of pulmonary metastases” was defined by the number of pulmonary nodules that were confirmed by the final pathologic report. Therefore, this number was not defined by the number of nodules actually resected or by the number of nodules that were presumed to be metastases by radiographic studies.

The following parameters were recorded and included in the statistical analysis: sex, age, number of resected pulmonary metastases, disease-free interval (DFI) between primary cancer resection and identification of pulmonary metastases, bilaterality, primary tumor site, surgical approach (thoracotomy, sternotomy, video-assisted thoracic surgery (VATS)), type of surgical resection (precision excision, wedge resection, segmentectomy, lobectomy, bilobectomy, or pneumonectomy), thoracic lymph node involvement, and administration of adjuvant chemotherapy.

The location of primary colorectal cancer was defined as two groups (the colon cancer group and the rectal cancer group) based on the anatomy of venous drainage. Patients were defined to have colon cancer if the primary lesion was between the cecum and rectosigmoid junction. In contrast, if the primary lesion fell between the rectosigmoid junction and the anus, patients were defined as having rectal cancer. Cancers with a distal margin at the anal verge or within 15 cm of it were classified as rectal cancer (when the exact anatomical location was not specified on the surgical report).

Our study was approved by the Institutional Review Board of Samsung Medical Center (IRB Number: 2015-01-089).

### Statistical analysis

For comparisons of continuous and categorical variables, either the Mann–Whitney *U* test, the *χ*^2^ test, or the Fisher exact test was used as appropriate. The Kaplan–Meier method was used to plot patient survival and recurrence curves. The following factors were evaluated for their influence on patient survival after pulmonary metastasectomy: age, gender, maximal tumor size (largest diameter in centimeters and/or the size of the largest metastasis), bilaterality, thoracic lymph node involvement, number of pulmonary metastases, and preoperative/adjuvant chemotherapy. Kaplan–Meier estimates and the log-rank test were used to calculate survival from the time of the first pulmonary metastasectomy to the last follow-up. A Cox proportional hazards model was used in univariate and multivariate analysis for these factors. *p* values <0.05 were considered statistically significant. All statistical analyses were performed using JMP version 11.0.1 (SAS Institute Inc., Cary, NC, USA).

## Results

### Patient demographics

A total of 696 patients underwent pulmonary metastasectomy with curative intent for CRC between November 1994 and December 2013. Among them, 81 patients had incomplete follow-up radiologic information and were therefore excluded. The remaining 615 patients were included in our study.

Patient characteristics according to the number of pulmonary metastases are summarized in Table 1. The median patient age at the time of pulmonary metastasectomy was 60 (range 26–82 years), and 361 patients (58.7 %) were male. The median follow-up period was 31.0 months (range 2–211 months). The median disease-free interval between the CRC surgery and pulmonary metastasectomy was 20 months (range 0–209). There were 244 primary cancers (39.7 %) in the colon and 371 in the rectum (60.3 %). Of all 615 patients, 370 (54.7 %) had received chemotherapy before pulmonary metastasectomy, and 461 (75.0 %) received adjuvant chemotherapy. Patients in groups II and III received more adjuvant chemotherapy than did those in group I (Table 1).

**Table 1:** Patient characteristics by the number of pulmonary metastases

Characteristics	The number of pulmonary metastases				*p* value^a^
	All patients	Group I	Group II	Group III	
	*N* = 615	*N* = 414	*N* = 159	*N* = 42	
Age, mean ± sd (years)	58.7 ± 10.4	59.3 ± 10.6	57.5 ± 10.2	57.5 ± 9.2	0.109
Male, *n* (%)	361 (58.7)	256 (61.8)	82 (51.6)	23 (54.7)	0.073
DFI, median (range), months	20 (0–209)	22 (0–209)	19 (0–112)	15 (0–108)	0.084
Primary CRC location					0.757
Colon cancer, *n* (%)	244 (39.7)	189 (45.6)	77 (48.4)	18 (42.9)	
Rectal cancer, *n* (%)	371 (60.3)	225 (54.4)	82 (51.6)	24 (57.1)	
Preop chemotherapy, *n* (%)	370 (54.9)	241 (58.2)	103 (64.8)	26 (61.9)	0.343
Adjuvant chemotherapy, *n* (%)	461 (75.0)	296 (71.5)	130 (81.7)	35 (83.3)	0.014

*DFI* disease-free interval (time between CRC surgery and pulmonary metastasectomy)

^a^Correlation between factors was assessed using *χ* ^2^ test and Student’s *t* test for continuous variables

### The number of lung metastases

**Fig. 1:**
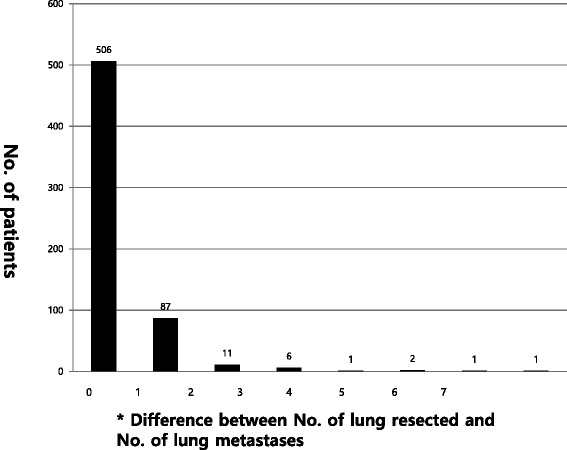
The difference between the number of lungs resected and the number of metastases

The mean number of lungs resected was 1.92 ± 1.77. The mean number of lung metastases was 1.66 ± 1.44. In 506 patients (82.3 %), every suspicious lung nodule that was resected was confirmed by pathology to be metastatic disease. However, in 109 patients (17.7 %), non-metastatic lesions (previously thought to be metastases) were resected (Fig. 1). Group I (*n* = 414) consisted of patients with a single pulmonary metastasis. Group II (*n* = 159) was made up of patients with two or three pulmonary metastases, and group III (*n* = 42) included patients with four or more pulmonary metastases. The mean number of metastases in groups I, II, and III were 1, 2.28 ± 0.45, and 5.79 ± 2.62, respectively (group I vs. II—*p* < 0.001; group II vs. III—*p* < 0.001).

### Surgical approach

The approach and extent of pulmonary metastasectomy is summarized in Table 2. The median size of the largest nodule was 1.3 cm (range 0.2–9 cm). There was no significant difference in tumor size between the three groups (*p* = 0.253). The majority of surgeries were performed using wedge resection (*n* = 439, 71.4 %), followed by lobectomy (*n* = 109, 17.7 %), segmentectomy (*n* = 41, 6.7 %), precision excision (*n* = 20, 3.3 %), bilobectomy (*n* = 5, 0.8 %), and pneumonectomy (*n* = 1, 0.2 %). With regard to surgical approach, 371 patients (60.3 %) underwent VATS, while the remaining 244 patients (39.7 %) had thoracotomy or sternotomy. Thoracotomy and sternotomy were performed more frequently in groups II and III than they were in group I (Table 2).

**Table 2:** Surgical management and pathologic findings by the number of pulmonary metastases

Characteristics	The number of pulmonary metastases				*p* value^a^
	All patients	Group I	Group II	Group III	
	*N* = 615	*N* = 414	*N* = 159	*N* = 42	
No. of lungs resected^b^, mean ± sd	1.92 ± 1.77	1.17 ± 0.46	2.64 ± 1.08	6.52 ± 3.31	<0.001
No. of lung metastases^c^, mean ± sd	1.66 ± 1.44	1.0	2.28 ± 0.45	5.79 ± 2.62	<0.001
Difference between no. of lungs resected and no. of metastases^d^, mean ± sd	0.25 ± 0.70	0.17 ± 0.46	0.33 ± 0.83	0.74 ± 145	<0.001
Maximum tumor size, mean ± sd	1.68 ± 1.23	1.64 ± 1.20	1.80 ± 1.34	1.67 ± 0.95	0.253
Bilaterality, *n* (%)	90 (14.6)	0	63 (39.6)	27 (64.3)	<0.001
Lymph node metastasis, *n* (%)^e^					0.614
No lymph node dissection	439 (71.4)	303 (73.2)	108 (67.9)	28 (66.7)	
Lymph node dissection	176 (28.6)	111 (26.8)	51 (32.1)	14 (33.3)	
LN (−)	152 (24.7)	95	44	13	
LN (+)	24 (3.9)	16	7	1	
Extent of resection, *n* (%)					0.442
Precision excision	20 (3.3)	17	3	0	
Wedge resection	439 (71.4)	300	111	28	
Segmentectomy	41 (6.7)	24	15	2	
Lobectomy	109 (17.7)	71	26	12	
Bilobectomy/pneumonectomy	6 (0.9)	2	4	0	
Surgical approach					<0.001
Thoracotomy/sternotomy	244 (39.7)	118 (28.5)	93 (58.5)	33 (78.6)	
VATS	371 (60.3)	296 (71.5)	66 (41.5)	9 (21.4)	

^a^Correlation between factors was assessed using *χ* ^2^ test and Student’s *t* test for continuous variables

^b^No. of lungs resected was defined as the number of pulmonary nodules resected in the operating room, not the number of pathologically confirmed metastatic lesions

^c^No. of lung metastases was defined as the number of pulmonary nodules that were pathologically confirmed to be metastatic cancer from CRC

^d^Difference between no. of lungs resected and no. of lung metastases = (no. of lung resected) − (no. of lung metastases)

^e^Definitions: LN (+), thoracic lymph node dissection was performed, and at least one lymph node was positive for malignant cells; LN (−), thoracic lymph node dissection was performed, and at least one lymph node was negative for malignant cells; no lymph node dissection, thoracic lymph node dissection was not performed

### Thoracic lymph node metastasis

A total of 176 patients (28.6 %) underwent thoracic lymph node dissection, with a median of five nodes dissected (range 1–38). Among these, 24 patients (24/176, 13.6 %) had lymph node metastases. There was no significant difference in the prevalence of thoracic lymph node metastasis between the three groups (Table 2).

### Overall and recurrence-free survival based on the number of lung metastases

**Fig. 2:**
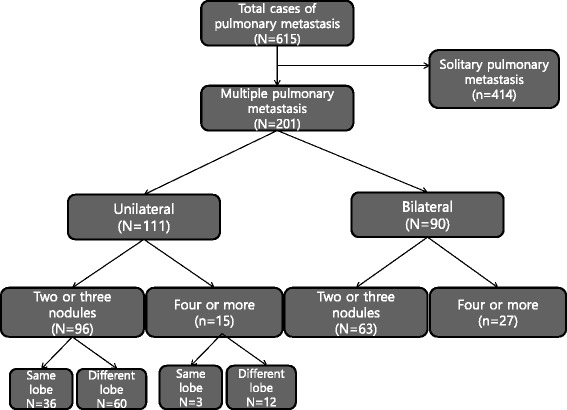
The number of patients according to the number of pathologically confirmed pulmonary metastases after pulmonary metastasectomy

**Fig. 3:**
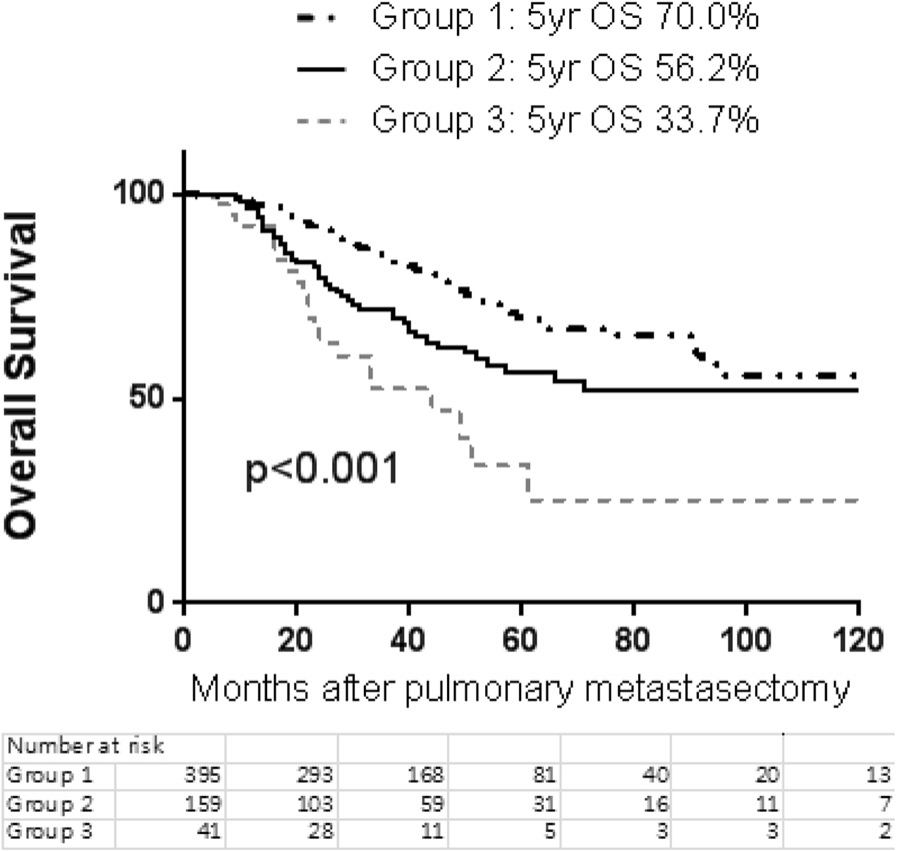
Overall survival from pulmonary metastasectomy according to the number of metastatic pulmonary nodules (group 1: single, group 2: 2–3 nodules, group 3: 4+ nodules)

**Fig. 4:**
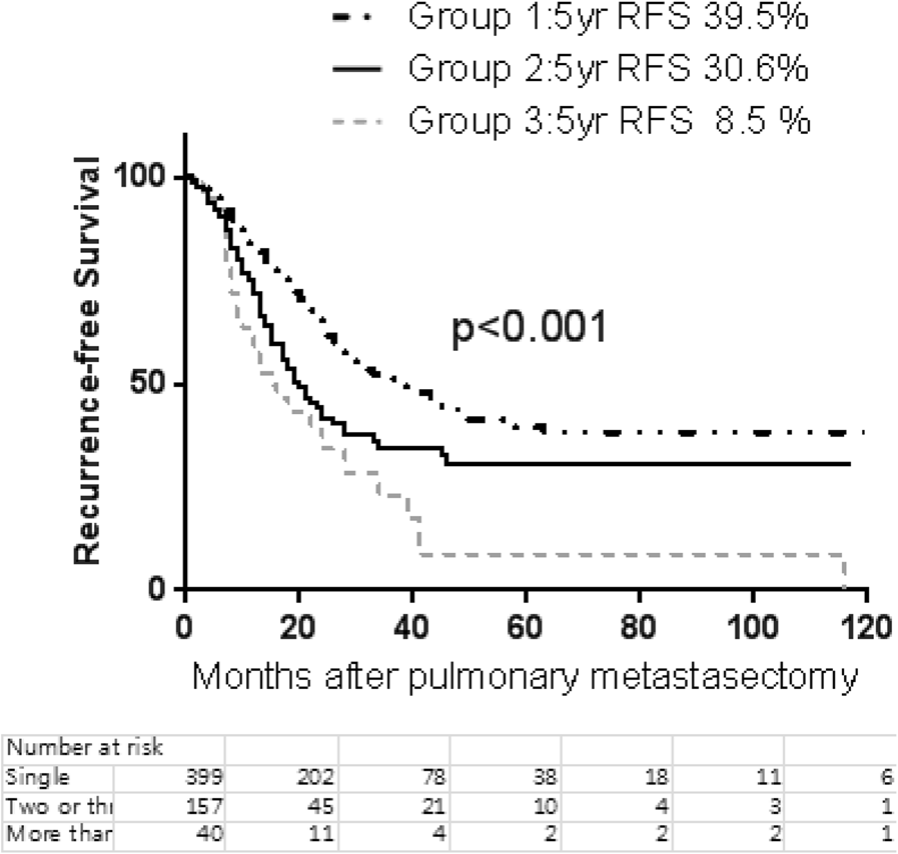
Recurrence-free survival from pulmonary metastasectomy according to the number of metastatic pulmonary nodules (group 1: single, group 2: 2–3 nodules, group 3: 4+ nodules)

The overall 5-year survival rate from pulmonary metastasectomy was 64.2 %. The recurrence-free survival rate was 34.8 % at 5 years. The CRC recurred after the treatment in 279 patients (45.4 %). The 5-year overall survival rates were 70.0, 56.2, and 33.7 % in groups I, II, and III, respectively (Fig. 2). There was a statistically significant difference in overall survival between the three groups (group I vs. II, *p* < 0.001; group II vs. III, *p* = 0.012). The 5-year recurrence-free rates were 39.5, 30.6, and 8.5 % in groups I, II, and III, respectively (Fig. 3). There was a statistically significant difference in recurrence-free survival between the three groups in terms of the number of pulmonary metastases (group I vs. II, *p* < 0.001; group II vs. III, *p* = 0.056) (Fig. 4).

The results of multivariate analysis for overall survival and recurrence after pulmonary metastasectomy are summarized in Tables 3 and 4, respectively. Multivariate analysis revealed that age (>70 years), multiplicity, thoracic lymph node involvement, and adjuvant chemotherapy are all independent prognostic factors of survival after pulmonary metastasectomy. Multiplicity and disease-free interval are independent prognostic factors that affect disease recurrence after pulmonary metastasectomy.

**Table 3:** Multivariate analysis of factors associated with overall survival from pulmonary metastasectomy

Variables	Hazard ratio	95 % confidence interval	*p* value
Age, years			
>70 vs. ≤70	1.95	1.22 ~ 3.02	0.007
Surgical approach			
Open surgery vs. VATS	1.39	0.97–1.99	0.070
Lymph node dissection			
LN (+) vs. no dissection	2.41	1.23–4.31	0.012
LN (+) vs. LN (−)	2.19	1.08–4.12	0.030
LN (−) vs. no dissection	0.63	0.74–1.61	0.627
Multiplicity			
4+ vs. single	4.42	2.50–7.49	<0.001
4+ vs. 2–3	2.25	1.31–3.75	0.003
2–3 vs. single	1.96	1.28–2.96	0.002
Adjuvant chemotherapy			
No vs. yes	1.77	1.25–2.47	0.002

Variables with a *p* value <0.10 were included in the multivariate analysis. Age is a continuous variable that was represented in groups for the Kaplan–Meier analysis. Definitions: LN (+), thoracic lymph node dissection was performed, and at least one lymph node was positive for malignant cells; LN (−), thoracic lymph node dissection was performed, and at least one lymph node was negative for malignant cells; no lymph node dissection, thoracic lymph node dissection was not performed. The Cox regression model was applied to identify risk factors for mortality and recurrence estimating the corresponding hazard ratios

**Table 4:** Multivariate analysis of the factors associated with recurrence-free survival from pulmonary metastasectomy

Variables	Hazard ratio	95 % confidence interval	*p* value
Disease-free interval, months			
≤36 vs. >36	1.51	1.14 ~ 2.04	0.004
Surgical approach			
Open surgery vs. VATS	1.22	0.95 ~ 1.57	0.115
Multiplicity			
4+ vs. single	2.17	1.29 ~ 3.52	0.004
4+ vs. 2–3	1.43	0.90 ~ 2.22	0.123
2–3 vs. single	1.51	1.10 ~ 2.05	0.012
Bilaterality			
Yes vs. no	1.00	0.67 ~ 1.49	0.990

Variables with *p* values <0.10 were included in the multivariate analysis. Disease-free interval is a continuous variable that was represented in groups for the Kaplan–Meier analysis. The Cox regression model was applied to identify risk factors for mortality and recurrence estimating the corresponding hazard ratios

#### Unilateral and bilateral metastases in patients with multiple lesions

Among those with multiple pulmonary nodules (*n* = 201), 90 patients had bilateral pulmonary metastases (Fig. 2). Eighty-six of these 90 patients (95.6 %) underwent a one-stage surgery including sternotomy, bilateral VATS, or thoracotomy (Table 5). The other four patients (4.4 %) had two-stage operations (Table 5). In patients with multiple metastases, there were no significant differences in overall survival (log rank; *p* = 0.666) and recurrence-free survival (log rank; *p* = 0.330) between unilateral and bilateral lesions.

**Table 5:** Surgical approach in patients with bilateral pulmonary metastases

Surgical approach	No. of patients	Group 2	Group 3
One-stage	86	59	27
Sternotomy	13	9	4
Bilateral VATS	29	23	6
Bilateral thoracotomy	44	27	17
Two-stage	4	4	0
Bilateral VATS	2	2	
Bilateral thoracotomy	2	2	
Total	90	63	27

Group 2: 2–3 pulmonary metastases; group 3: 4+ pulmonary metastases

## Discussion

Many prior studies have suggested that the number of pulmonary metastases is an important prognostic factor of survival and recurrence after pulmonary metastasectomy from CRC [7–14]. The current surgical strategy regarding pulmonary metastasectomy is to resect every pulmonary metastases, regardless if a patient has one or multiple lesions [15, 16]. Previously, there had been no defined cutoff number of lung metastases that would prevent possible treatment by metastasectomy. Only a few reports had showed that patients with many pulmonary nodules have a poor prognosis after pulmonary metastasectomy. Onaitis et al. showed that having more than three pulmonary metastases (from CRC) was an independent poor prognostic factor of survival after pulmonary metastasectomy [17]. Similarly, Blackmon et al. found more than three metastases predicted poor prognosis for survival and recurrence after metastasectomy [18]. Our data reveal that the most potent predictor of survival and recurrence is the number of metastases. Patients with a single metastasis have over fourfold better survival than do those patients with four or more metastases. Patients who had four or more pulmonary metastases were more than twice as likely to experience a recurrence than those patients with a single metastasis. The 5-year recurrence-free survival rate in patients with 4+ pulmonary metastases was only 8.5 %. Therefore, patients with four or more pulmonary metastases might not benefit from pulmonary metastasectomy. A meticulous selection process and multidisciplinary approach is indicated to decide treatment modalities for such patients. However, it is still uncertain whether other treatment modalities such as chemotherapy or radiotherapy are superior to surgery in patients with multiple pulmonary metastases. Therefore, it is important to emphasize that having multiple (4+) pulmonary metastases does not rule out the possibility of using pulmonary metastasectomy in a patient’s treatment. Surgery may still be considered if the patient is young, has good performance status, has adequate pulmonary function, has no severe comorbidities, and has no evident of thoracic lymph node metastasis on chest CT or PET-CT. In our study, even patients with multiple (4+) pulmonary metastases had >30 % 5-year overall survival rate after pulmonary metastasectomy. It is well known that the median survival for stage IV CRC is only 5–6 months, if left untreated [19].

It is important to recognize that patients thought to have solitary lesions may have also had occult micrometastases that were undetected, resulting in incomplete resection. Cerfolio et al. [20] found that during metastasectomy, non-imaged malignant pulmonary metastases are found in 18 % of patients who have had a previously treated solid organ cancer and at least one imaged metastatic pulmonary lesion. Therefore, a solitary lung metastasis may actually be one of the several unidentified lesions. On the other hand, pulmonary nodules that are recognized as metastases by CT scanning are not always true metastatic disease. Inflammatory lesions or intrapulmonary lymph nodes can be mistaken for cancer in the lung. In 109 patients (17.7 %), non-metastatic lesions were resected during surgery, in addition to truly metastatic nodules. Although this involves removing some normal tissue, we believe that it is safer to remove all suspicious pulmonary nodules than it is to risk missing lesions that are actually malignant.

Besides multiplicity, we also found that patients with a shorter DFI (<36 months) had high rates of recurrence after surgery. Patients with DFI <36 months had a 1.51-fold increased risk of recurrence compared to those with longer DFI. However, multivariate analysis did not identify DFI as an independent prognostic factor of survival after pulmonary metastasectomy.

A multidisciplinary approach is indicated when pulmonary metastasectomy is being considered in cases with multiple lesions. In the case of a single metastasis, the surgical approach is aggressive as long as resectability is confirmed. However, in patients with multiple nodules, the risk of surgical comorbidities may be higher than it is in patients with a single metastasis. Interestingly, our data did not show a significant survival difference between patients with unilateral and bilateral lesions. In the present study, there was no significant difference in outcome between patients with bilateral metastases compared to those with multiple unilateral lesions. There are conflicting data regarding whether the distribution of metastases affects patient survival after pulmonary metastasectomy [9, 21, 22]. Bilaterality is not an absolute contraindication to metastasectomy as long as the lesions can be completely resected. However, a careful approach is important in cases of bilateral pulmonary metastases or involvement of multiple unilateral lobes. Although we did not address the issue of repeated metastasectomy, it likely carries a higher risk of morbidity than does a single procedure. Therefore, patients requiring repeated metastasectomy should be chosen carefully after meticulous evaluation.

A few prior reports have suggested that mediastinal lymph node involvement is a prognostic factor of pulmonary metastasectomy success. Thoracic lymph node metastasis, which occurs in 12–19.2 % of patients with CRC, is recognized as a poor prognostic factor [23–25]. In this study, 13.6 % (24/176) of patients had thoracic lymph node metastases. The Cox proportional hazards method revealed that thoracic lymph node metastasis was a significant factor of overall survival. This finding is consistent to that of Hamaji et al. [26], who also found that thoracic lymph node metastasis is a negative prognostic factor. However, as the group noted, it is unknown whether thoracic or mediastinal lymph node dissection has any therapeutic effect. Systematic lymph node dissection is currently offered routinely for prognostic purposes, but not yet for therapeutic ones.

This study has several limitations. Patients were enrolled from a single institution, and the data were retrospectively reviewed. Furthermore, follow-up was not completed in all patients, and in most patients, the recurrence rates after pulmonary metastasectomy were defined based on radiologic findings. Despite these limitations, our data suggest that the surgical strategy for pulmonary metastasectomy should be tailored to the patient depending on the number of pulmonary metastases.

## Conclusions

The overall survival and recurrence after pulmonary metastasectomy for CRC is dependent on the number of metastases. Surgical treatment can be offered to patients with three or fewer pulmonary metastases. However, more meticulous patient selection is required to decide whether a surgical approach is feasible in patients with four or more pulmonary metastases.
